# Basic Research on the Preparation of Electrolytic Manganese Residue–Red Mud–Ground Granulated Blast Furnace Slag–Calcium Hydroxide Composite Cementitious Material and Its Mechanical Properties

**DOI:** 10.3390/ma18061218

**Published:** 2025-03-10

**Authors:** Biao Peng, Lusen Wang, Zhonglin Li, Ye Xu, Weiguang Zhang, Yibing Li

**Affiliations:** Department of Materials Science and Engineering, Guilin University of Technology, Guilin 541004, China; pengbiao1220@163.com (B.P.); wls20000206@163.com (L.W.); xuye121306@163.com (Y.X.); zhangwg@glut.edu.cn (W.Z.)

**Keywords:** red mud, electrolytic manganese residue, green cementitious material, ground granulated blast furnace slag

## Abstract

A novel composite cementitious material was constructed by synergistically utilizing multiple industrial solid wastes, including electrolytic manganese residue (EMR), red mud (RM), and ground granulated blast furnace slag (GGBS), with calcium hydroxide [Ca(OH)_2_] as an alkaline activator. In addition, the mechanical properties of the composite cementitious materials were systematically analyzed under different raw material ratios, alkali activator dosages, and water-binder ratios. To further investigate the hydration products and mechanisms of the composite cementitious material, characterization methods, for instance, XRD, FT-IR, SEM-EDS, and TG-DTG, were employed to characterize the materials. To ensure that the composite cementitious material does not cause additional environmental pressure, it was analyzed for toxic leaching. The relevant experimental results indicate that the optimal ratio of the EMR–RM–GGBS–Ca(OH)_2_ components of the composite cementitious material is EMR content of 20%, RM content of 15%, GGBS content of 52%, calcium hydroxide as alkali activator content of 13%, and water-binder ratio of 0.5. Under the optimal ratio, the composite cementitious material at 28 days exhibited a compressive strength of 27.9 MPa, as well as a flexural strength of 7.5 MPa. The hydration products in the as-synthesized composite cementitious material system primarily encompassed ettringite (AFt) and hydrated calcium silicate (C-S-H), and their tight bonding in the middle and later curing stages was the main source of engineering mechanical strength. The heavy metal concentrations in the 28-day leaching solution of the EMR–RM–GGBS–Ca(OH)_2_ composite cementitious material fall within the limits prescribed by the drinking water hygiene standard (GB5749-2022), indicating that this composite material exhibits satisfactory safety performance. To sum up, it is elucidated that the novel process involved in this research provide useful references for the pollution-free treatment and resource utilization of solid wastes such as red mud and electrolytic manganese residue in the future.

## 1. Introduction

In the past few years, China’s accelerated technological advancement and industrial upgrading in metallurgical production systems has led to an increase in the emissions of industrial solid wastes, such as manganese electrolysis residue and red mud [1]. Among them, electrolytic manganese residue (EMR), an acidic solid waste residue, is produced by the industrial production of manganese metal using electrolysis, which generates 8–12 tons of EMR per ton of manganese metal [2,3]. When using the Bayer process to produce alumina in industry, a byproduct is obtained, which is alkaline, and we call it red mud (RM). The composition of RM particularly depends on the characteristics of raw ore and metallurgical process flow [4], and it is estimated that annual production of RM is up to 120 million tons [5]. These industrial solid wastes have complex compositions, and most of them contain heavy metal elements such as Zn, As, Gr, or other environmentally hazardous chemical substances [6], which can cause serious harm to the ecological environment if improperly disposed of [7,8,9,10]. This type of treatment of industrial solid waste can cause serious resource waste and environmental damage. To this end, we should implement the idea of ecological civilization of “resourcefulness, lightness and harmlessness” to minimize the negative impact of metallurgical solid waste on the ecological environment [11].

RM and EMR are different from other industrial solid wastes. RM contains volcanic ash reactive minerals (Al_2_O_3_ and SiO_2_, etc.), which proves that it possesses the potential ability to be used to prepare cementitious materials for practical civil engineering [12]. Koshy et al. found that soluble bases in red mud form an alkaline environment when dissolved in water and can provide some assistance in the alkaline environment required for the formation of cementitious materials [13,14]. Li et al. prepared ternary cementitious materials using red mud, lead/zinc smelting waste as raw materials, and calcium oxide as the main material. After 28 days of curing, the compressive strength was 12.05 MPa [15]. Li et al. used sodium hydroxide to provide an alkaline environment and prepared binary cementitious materials using red mud and waste glass sludge as raw materials. The related experimental data revealed that the cementitious material, after 28 days of curing, exhibited a compressive strength of 15.98 MPa [16]. Cui et al. prepared quaternary ternary cementitious materials using RM, steel slag, fly ash, and phosphatic lime as the main materials and found that the compressive strength was 21.4 MPa [17]. Although there have been many applications of RM in the field of cementitious materials, the strength of the cementitious materials prepared from red mud is slightly insufficient. Sulfates contained in EMR can be used as active agents in materials such as mineral powders to prepare composite cementitious materials [18]. Wu et al. used blast furnace slag, coal slag, and electrolytic manganese residue as raw materials and sodium hydroxide as an alkali exciter to prepare ternary cementitious materials, curing electrolytic manganese residue within the harmful elements to reduce the hazards to the environment, with a compressive strength of 22.8 MPa [19]. Li et al. prepared an EMR-FA binary cementitious material using a composite solution composed of sodium hydroxide and sodium silicate, incorporating electrolytic manganese residue and fly ash as raw materials; after curing for 28 d, the compressive strength of the binary cementitious material was 15.6 MPa [20]. At present, When using EMR as raw materials to prepare cementitious materials without adding silicate cement, their compressive strength can only reach around 20 MPa. He et al. added Portland cement to EMR-based cementitious materials, which resulted in a 28-day compressive strength of 44 MPa [21]. Although achieving good physical properties, the addition of cement actually goes against our original intention of solid waste resource utilization. Many researchers are committed to replacing cement with other volcanic ash active solid waste to achieve better results [22,23].

The use of RM, EMR, GGBS, and slaked lime as raw materials for the preparation of cementitious materials increases consumption of these solid waste to avoid the harm brought to the ecological environment, but also enhances the physical properties of cementitious materials to meet the mechanical properties required for civil engineering applications. Hence, a novel composite cementitious material was constructed by synergistically utilizing multiple industrial solid wastes, including electrolytic manganese residue (EMR), red mud (RM), and ground granulated blast furnace slag (GGBS), with calcium hydroxide [Ca(OH)_2_] as an alkaline activator. Characterization tools such as XRD, FT-IR, SEM-EDS, and TG-DTG were employed to deeply analyze the hydration products of the cementitious materials and reveal the hydration mechanism. Moreover, toxicity leaching tests were conducted to assess their impact on the environment, thus providing a useful reference for the future harmless treatment and resource utilization of solid wastes such as red mud and electrolytic manganese residue in the future.

## 2. Reaction Mechanism of Geopolymers

Geopolymers are inorganic polymeric materials rich in aluminum and silicon in alkaline environments, consisting of a three-dimensional network of AlO_4_ and SiO_4_ tetrahedral structures [24]. The formation process of geopolymer gels is divided into the following stages: (1) dissolution stage: high-energy aluminum and silicon bonds in the raw material are broken under the action of OH^−^ in alkali exciters (KOH, NaOH, etc.); (2) depolymerization stage: dissolved Al^3+^ and Si^2+^ form aluminates and silicates with metal ions in an alkaline environment; (3) polymerization stage: as the reaction proceeds, the depolymerization stage forms aluminates and silicates, gradually forming a three-dimensional network gel.

For the EMR–RM–GGBS–Ca(OH)_2_ composite cementitious material, in the first stage the Al-O and Si-O in the red mud and GGBS fracture in the presence of alkali exciters to form [H_3_SiO_4_]^−^, [H_3_AlO_4_]^2−^, and [Al(OH)_6_]^3−^, which are dissolved in the aqueous phase [25].

In the second stage, the [H_3_SiO_4_]^−^ and [H_3_AlO_4_]^2−^ of the tetrahedral structure in the aqueous solution gradually combine with the calcium ions in solution to form gelling substances, including C-A-S-H and C-S-H. Meanwhile, the hexahedral [Al(OH)_6_]^3−^ in solution forms calcite crystals in the presence of sulfate in the electrolytic manganese residue and the alkali exciter calcium hydroxide [26].

Calcite crystals fill in the 3D network structure of the gel material, making the structure denser and improving the strength [27]. As the reaction continues, the water phase in the system continues to decrease, and the calcium-containing gels in the system are cured by mutual gelation polymerization.

## 3. Experimental Design

### 3.1. Raw Materials

The raw materials were characterized as follows: (1) red mud (RM), sourced from a metallurgical materials company in Fangchenggang, China, represents a Bayer process–derived alkaline residue exhibiting reddish-brown lumpy morphology with a measured pH of 10.35; (2) electrolytic manganese residue (EMR), obtained from a manganese mining branch in Guilin, China, manifests as black compacted plate-like solids with a measured pH of 6.45; (3) GGBS is S95 standard slag powder from a company in Gongyi, China; calcium hydroxide (analytically pure) was purchased from Xilong Chemical Co., Ltd. (Chaoshan, China).

**Figure 1:**
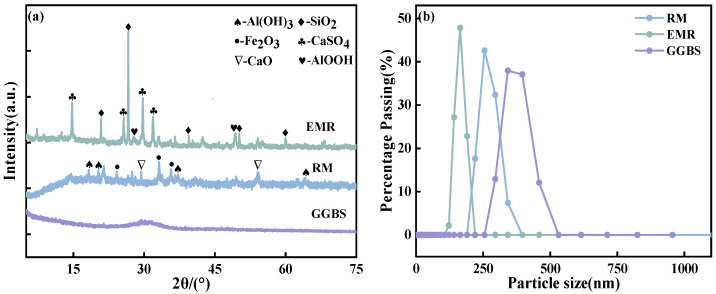
(**a**) Raw material XRD; (**b**) raw material particle size.

Figure 1a is an XRD diagram of the raw material. The physical phase in the raw material was analyzed. The composition of the physical phase of GGBS is not obvious, which indicates that it contains a large amount of glassy material with good volcanic ash activity. Both RM and EMR have a certain amount of aluminum, which is one of the most important elements for the construction of this novel type of geopolymer. Figure 1b shows the distribution of the particle size and indicates that the particle size of RM is mainly in the range of 200–400 nm, while that of EMR is in the range of 100–250 nm. The particle size of GGBS is slightly larger than that of red mud and electrolytic manganese slag, with a particle size distribution in the range of 250–550 nm.

The oxide compositions of RM, EMR, and GGBS were analyzed using an XRF fluorescence spectrometer (Zetium, Panaco, The Netherlands) and are presented in Table 1. The primary elements that play a role in the geopolymerization process are Ca, Si, and Al, as they constitute the main components of ettringite (AFt) and C-A-S-H gel. The data in Table 1 show that GGBS is a high-quality material that provide sufficient Ca, Si, and Al. RM mainly provides Al, and EMR can provide Ca and Si, and SO_4_^2−^ in the EMR can promote the generation of AFt.

**Figure 2:**
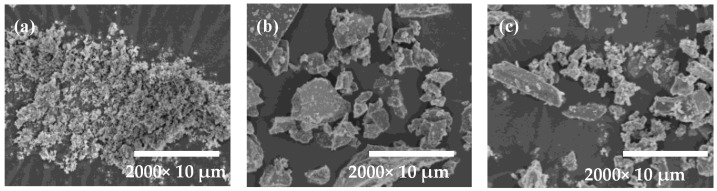
SEM images of raw materials: (**a**) RM, (**b**) GGBS, and (**c**) EMR.

SEM analysis of the raw material was carried out, as shown in Figure 2. RM is irregular and granular, with flaky particles embedded in it; GGBS is irregularly sized lumpy particles; EMR is composed of columnar particles and irregular fine particles.

### 3.2. Mix Proportions and Preparation

**Figure 3:**
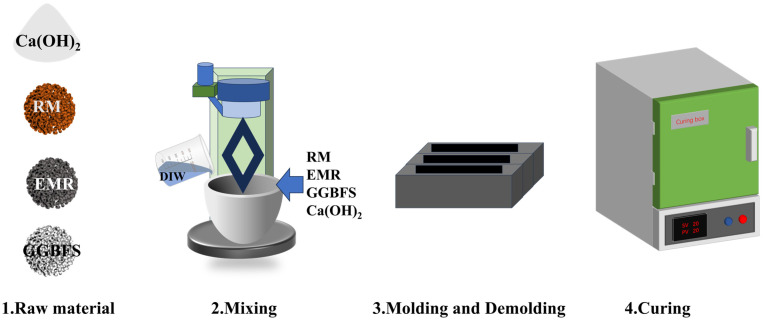
Composite cementitious material mortar preparation process.

Preparation process of the EMR–RM–GGBS–Ca(OH)_2_ composite cementitious material: pour the dried EMR, RM, GGBS, and Ca(OH)_2_ into the mortar mixer and stir for 200–300 s to achieve homogeneous powder dispersion. After all the ingredients have been mixed well, continue mixing for about 60–90 s after adding a quantitative amount of tap water. Then pour the mixed paste into the molds, demold after 24 h of curing, and place into the standard curing chamber for curing (shown in Figure 3).

To study the optimal mix proportion of the EMR–RM–GGBS–Ca(OH)_2_ composite cementitious material for superior mechanical performance, following determination of the optimal formulation, a systematic examination of its mechanical properties under varying water-binder ratios was conducted. The raw material ratios and water-binder ratios of the as-constructed composite cementitious material are shown in Table 2. The size of the test block was 40 × 40 × 160 mm, and ISO standard sand was used to mold, cure, and test its mechanical properties with reference to the Test Methods for Cement Mortar Strength (ISO Method) [28]. The 40 × 40 × 40 mm cubic net mortar specimens were prepared with the same curing conditions as the mortar specimens, broken after 3, 7, and 28 days of curing, immersed in anhydrous ethanol for 24 h to terminate the hydration reaction, and preserved by low-temperature drying for microanalysis.

### 3.3. Testing Items and Methods

#### 3.3.1. Physical and Mechanical Properties

The EMR–RM–GGBS–Ca(OH)_2_ composite cementitious materials were prepared and cured in accordance with the Test Methods for Cement Mortar Strength (ISO Method), and their compressive and flexural strengths were tested at the corresponding ages (3, 7, and 28 days). In order to reduce the chance of error, each series of each age of curing were prepared in triplicate to measure the average value of three specimens as the determination of the value.

#### 3.3.2. Microstructure Characterization

A field emission scanning electron microscope (Hitachi, Tokyo, Japan) was employed to further investigate the surface microstructure of the EMR–RM–GGBS–Ca(OH)_2_ composite cementitious materials at 3, 7, and 28 days of curing. At the same time, EDS was used to analyze the concentration of chemical elements in the composite cementitious material and hydration products. The composition phases of the raw materials and the EMR–RM–GGBS–Ca(OH)_2_ materials were analyzed via an X-ray diffraction analyzer (PANalytical B.V., Almelo, The Netherlands). Moreover, Fourier transform infrared spectroscopy (Thermo Fisher Scientific, Waltham, MA, USA) was conducted to explore the chemical structure of the hydration products of the EMR–RM–GGBS–Ca(OH)_2_ materials. The samples were analyzed by TG-DTG (NETZSCH, Selb, Germany) using a synchronous thermal analyzer under nitrogen, the temperature increase rate and test temperature range were set to 10 °C/min and 20–1000 °C, respectively. The concentration of heavy metals in the leachate of the test blocks was measured by means of inductively coupled plasma emission spectroscopy.

#### 3.3.3. Solidification Time Test

The initial and final setting times of the EMR–RM–GGBS–Ca(OH)_2_ composite cementitious materials were tested using a Vicat apparatus. The mixed slurry was poured into the molds used for testing, and as much air as possible was expelled from the slurry. The priming needle was then mounted on the Vicat apparatus; when it contacted the glass plate, the pointer was adjusted to align with the zero mark. When testing the initial coagulation needle to contact the surface of the slurry, it was allowed to slowly decline until the initial coagulation needle was no longer falling, and the pointer showed 4 mm ± 1 mm when the initial coagulation state was reached. The initial coagulation needle was replaced with the final coagulation needle, and the mold was turned over, so that the final coagulation needle contact with the surface of the cementitious material 30 s after the surface of the cementitious material only made 0.5 mm deep pinholes and no ring marks. The cementitious material at this time had reached the final state.

## 4. Results and Discussion

### 4.1. Effect of Various RM Dosages on Mechanical Strength

**Figure 4:**
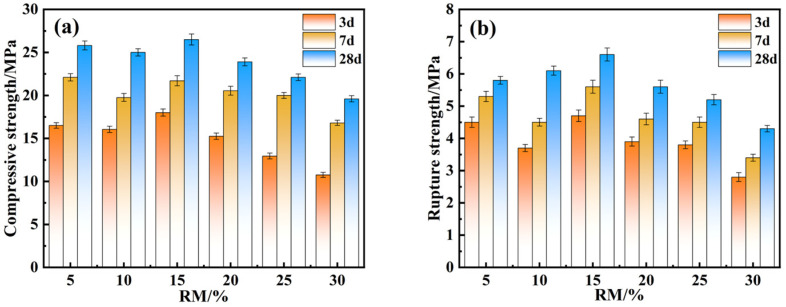
Effect of various RM dosages on compressive strength (**a**) and rupture strength (**b**) of the EMR–RM–GGBS–Ca(OH)_2_ materials.

Figure 4 illustrates the effect of various RM dosages on the mechanical strength of the EMR–RM–GGBS–Ca(OH)_2_ composite cementitious materials. The compressive strength of the specimens exhibited a tendency of decreasing and then increasing. The mechanical properties of samples with RM dosages of 5, 10, and 15 were 25.8 MPa, 25 MPa, and 26.5 MPa, respectively. The excessive RM dosage of 15% inhibited compressive strength. Additionally, the flexural strength exhibited an increasing and then decreasing tendency; the flexural strength was 5.8 MPa, 6.1 MPa, and 6.6 MPa with the various red mud dosages of 5%, 10%, and 15%, respectively. When the addition of RM was over 15%, the flexural strength of the EMR–RM–GGBS–Ca(OH)_2_ composite cementitious materials decreased gradually. RM is alkaline solid waste. When the red mud addition was less than 15%, under the condition of fixing the amount of alkali exciter, the increase in RM made the alkali concentration in the composite gelling system increase, which promoted the geological polymerization reaction and generated more gelling material. The primary component of RM is hematite (Fe_2_O_3_), in which the silica–aluminum component accounts for less [29], and the degree of activity of RM is much smaller than that of GGBS. Furthermore, the increase of red mud dosage leads to an increase in inert components of the system; the active silica–aluminum component was inhibited in the reaction system involved in the hydration reaction, resulting in a reduction in the generation of gelling material, which led to an inhibition in the mechanical properties. To summarize, an RM dosage of 15% was determined to be the optimal proportion.

### 4.2. Effect of Alkali Exciter Dosage on Mechanical Properties

**Figure 5:**
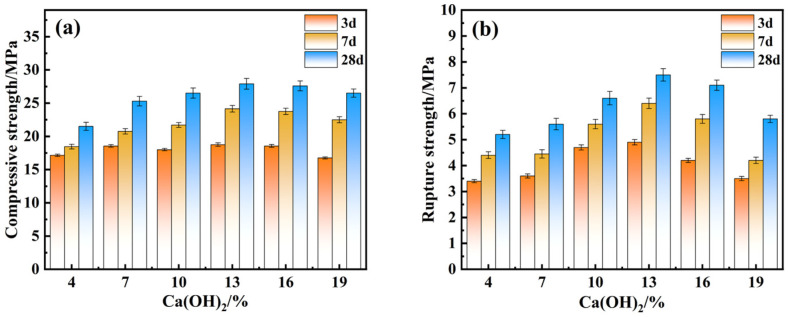
Effect of the amount of alkali exciter on the compressive strength (**a**) and rupture strength (**b**).

The alkali exciter Ca(OH)_2_ provides an abundance of OH^−^, which provides the alkalinity needed for the hydration reaction in the EMR–RM–GGBS–Ca(OH)_2_ cementitious material system and also provides the necessary calcium ions for formation of the EMR–RM–GGBS–Ca(OH)_2_ material. Figure 5 shows the effect of the amount of alkali exciter on the compressive and flexural strength of the composite cementitious material.

Figure 5 reveals that the compressive strength increased and then reduced with a continuous increase of Ca(OH)_2_ addition. At dosages of 13% and 16%, the difference in 28 d compressive strength was not large, at 27.9 MPa and 27.6 MPa, respectively, and as the dosage increased further, the compressive strength decreased. The rupture strength presented a trend of initially increasing and subsequently decreasing with the increase of Ca(OH)_2_ addition, and the highest rupture strength of 7.5 MPa was found with the addition of Ca(OH)_2_, as well as a flexural strength of 7.5 MPa. Within the lower dosage range, the OH^−^ in the material system enhanced with the increase in the amount of alkaline activator Ca(OH)_2_, and the hydration reaction in the material system was complete. The compressive strength started to decrease when the addition of alkali exciters exceeded 16%, while the rupture strength started to decrease when the dosage exceeded 13%. The increase in Ca(OH)_2_ doping made the Si and Al elements fully react to generate cementitious materials, and Ca^2+^ reacted with SO_4_^2+^ in the system to form AFt to make the structure more dense, thus providing increased strength. With the continuous addition of Ca(OH)_2_, the entire system has become excessively alkaline. Excess alkali overflowed with the water to the surface of the test piece to form a “white frost”. The formation of white frost involves the efflorescence behavior of geopolymers, which is mainly due to free alkali inside the system penetrating to the surface through the pore water and thus producing the weathering phenomenon. The emergence of these phenomenon due to alkali will destroy the internal structure in the EMR–RM–GGBS–Ca(OH)_2_ cementitious material system, ultimately resulting in a decline in compressive strength and rupture strength [30]. In summary, it is appropriate to control the dosage of the alkali exciter Ca(OH)_2_ at 13%.

### 4.3. Effect of Mechanical Properties of Water-Binder Ratio

**Figure 6:**
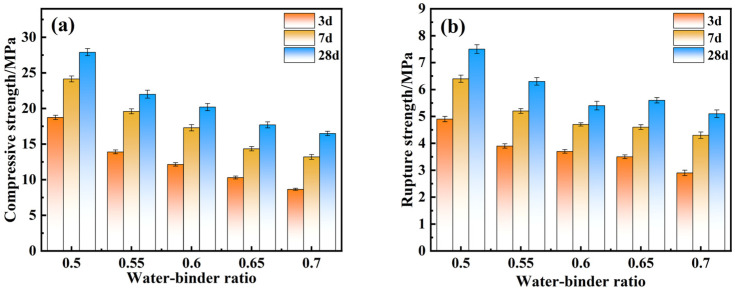
The compressive strength (**a**) and rupture strength (**b**) of composite cementitious materials with various water-binder ratios.

Water is essential in geopolymerization. Water not only makes the active Si and Al in the material fully dissolve in the pulp, but also promotes more complete reactions of depolymerization and polymerization in geopolymerization reactions. The ratio of the water-binder in the synthesis process exhibited a huge impact on the EMR–RM–GGBS–Ca(OH)_2_ composite cementitious system. Figure 6 shows the intrinsic influence of various water-binder ratios on the mechanics of the composite cementitious materials. With the increase in water-binder ratio, the compressive strength and rupture strength gradually decreased. When the water-binder ratio was lower than 0.5, the amount of water in the test process was too little, which made the material hard to mix and the solidification too fast, so the specimen could not be molded. When the water-binder ratio was greater than 0.5, the excess water made the alkali concentration in the composite cementitious system decrease, which hindered the hydration reaction. There will also be a part of the water will be attached to the surface of the hydration product, this part of the water will be in the subsequent maintenance process through the volatilization of the test specimen of the internal formation of pores, resulting in the structure is not dense, thus affecting the compressive strength and rupture strength. To summarize, the water-binder ratio was selected as 0.5.

Through the above experiments with different influencing factors, we determined the optimum proportion of composite cementitious materials and the optimum water-binder ratio. The required test blocks were prepared with EMR, RM, GGBS, and Ca(OH)_2_ at 20%, 15%, 52%, and 13% of the total material mass fraction, respectively, and a water-binder ratio of 0.5. In order to study the hydration products and hydration mechanism, we used various microscopic characterization means to characterize the prepared specimen blocks.

### 4.4. Physical Analysis of Hydration Products

**Figure 7:**
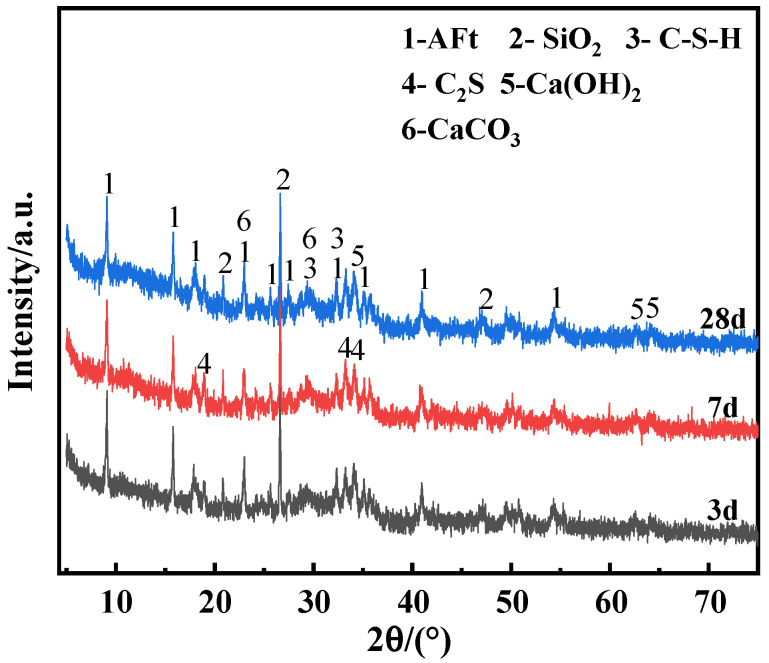
XRD patterns of net mortar specimens at different curing ages.

In order to investigate the hydration products of the EMR–RM–GGBS–Ca(OH)_2_ composite cementitious materials at different curing periods, XRD analysis was carried out. Figure 7 shows the XRD diagrams of the net slurry specimens at different curing ages, and it can be seen that there was not much difference in the physical phases of the specimens at different curing ages; only the intensity of the diffraction peaks had some changes. The main physical phases were ettringite (AFt), quartz (SiO_2_), calcite (CaCO_3_), calcium hydroxide [Ca(OH)_2_)], and hydrated calcium silicate (C-S-H). The presence of stabilized quartz in the physical phase can act as a filler in the system. The appearance of the characteristic peak of CaCO_3_ indicates that carbonization of the test block occurred during the conservation process, and Ca(OH)_2_ in the test block reacted with CO_2_ in the air to form CaCO_3_. The presence of characteristic peaks of calcium hydroxide in the physical phase after 28 d of curing indicates that the calcium hydroxide was not completely consumed. The diffraction peak of AFt appeared early in curing (within 3 d), and the generation of AFt built up the early strength of the specimen. With prolongation of the curing time, the characteristic peak intensity of Ca(OH)_2_ gradually decreased, while the diffraction peak intensity of AFt gradually strengthened. This is because the SO_4_^2−^ contained in the electrolytic manganese residue and the reactive silica–alumina component in the RM participated in the hydration reaction to consume part of the calcium hydroxide, and the alkali contained in the RM also promoted the hydration reaction [31], accelerating the generation of gelling substances such as AFt and C-S-H.

### 4.5. Chemical Bonding Analysis of Hydration Products

**Figure 8:**
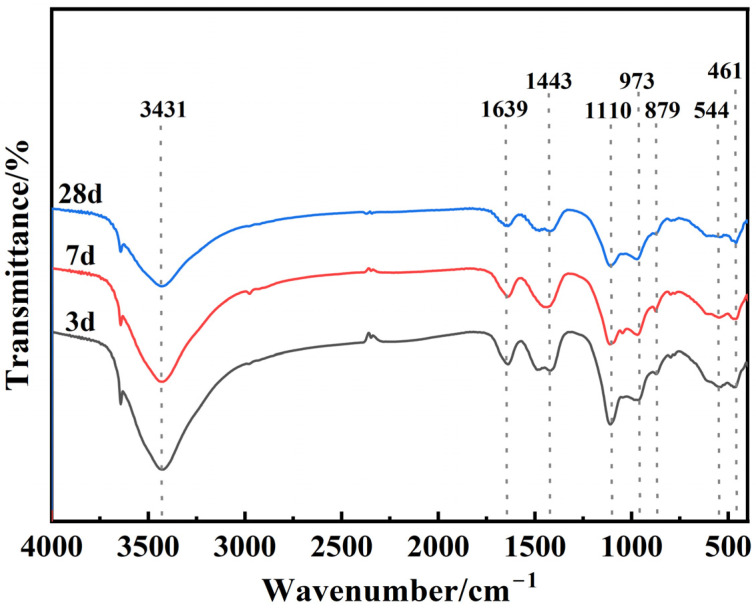
FTIR patterns of net mortar specimens at different curing ages.

Figure 8 shows the FT-IR spectra of the as-synthesized EMR–RM–GGBS–Ca(OH)_2_ composite cementitious materials at different curing stages. For specimens of different curing ages, there was a great difference in the peak absorption intensity at the same location. The bonds located at 3431 cm^−1^ and 1639 cm^−1^ belong to the bending and stretching vibrations of the O-H groups in AFt [32]. The contraction vibration of the S-O bonds at 1110 cm^−1^ suggests that SO_4_^2−^ is involved in the hydration reaction. The absorption bonds at 973 cm^−1^ are associated with the asymmetric telescopic vibration of Si-O-T (T=Si, Al), suggesting generation of a hydrated calcium silicate (C-S-H) hydration product. The characteristic peaks at 1445 cm^−1^ and 879 cm^−1^ are spectral peaks due to C-O vibration and CO_3_^2−^ deformation vibration in CaCO_3_ [33], which indicates that the specimen blocks reacted with CO_2_ in the air during the conservation process, resulting in carbonization. The bonds near at 544 cm^−1^ correspond to the absorption vibration of Al-O, and the bonds at 461 cm^−1^ c correspond to the bending vibration of Si-O, which may be related to quartz decomposition.

### 4.6. TG-DTG Analysis of Hydration Products

**Figure 9:**
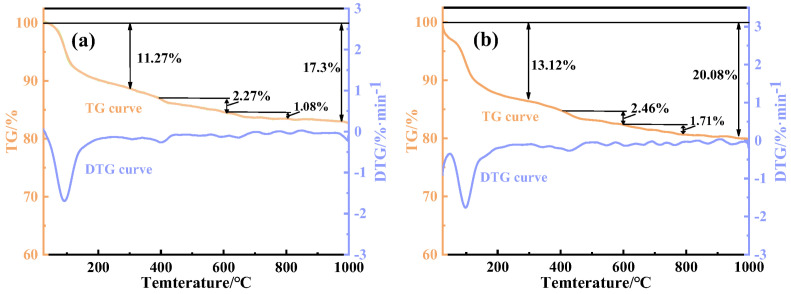
TG-DTG curves at 3 d (**a**) and 28 d (**b**) in the net mortar specimens.

Figure 9 presents the TG-DTG curves of the as-synthesized EMR–RM–GGBS–Ca(OH)_2_ composite cementitious materials at different curing stages (3 days and 28 days). As it shows, there were three weight loss stages. Combined with XRD and FTIR analyses, it shows that there are three weight loss stages, and the main weight loss stage for 3 d and 28 d of conservation is 30~300 °C, which is the thermal decomposition of calcium AFt and C-S-H gels [34]; 400~600 °C mass loss at this stage is Ca(OH)_2_ decomposition [35]; the 600~800 °C stage of mass loss is mainly for the specimen in the conservation process and the reaction of CO_2_ in the air to generate CaCO_3._ Thermal decomposition [36] caused by these two stages of weight loss is not obvious. The mass loss rate of the material after 3 days of curing is presented: the mass loss of the 30~300 °C temperature range was 11.27%, the mass loss of the 400~600 °C temperature range was 2.27%, the mass loss of the 600~800 °C temperature range was 1.08%, and the loss of the total mass of the samples was 17.3%. The mass loss rate of the material after 28 days of curing is presented: the mass loss of the 30~300 °C temperature range was 13.12%, the mass loss of the 400~600 °C temperature range was 2.46%, the mass loss of the 600~800 °C temperature range was 1.71%, and the loss of the total mass was 20.08%, which is higher than that of the material after 3 days of curing. This indicates that the content of AFt and C-S-H gel in the EMR–RM–GGBS–Ca(OH)_2_ composite cementitious system after 28 d of curing was more than that after 28 d of curing, which verifies that, as the curing time extends, the hydration reaction is continuously carried out in the system of composite cementitious materials, and the generation of more AFt and C-S-H gel is conducive to enhancement of the strength of the specimen [10], which is consistent with the relevant mechanical test and XRD analysis.

From the above analysis, it can be seen that the content of the hydration products AFt and calcium silicate hydrate (C-S-H) gel that accumulates during 28 d of curing is more than that produced during 3 d of curing, which indicates that, with the increase in curing time, the hydration reaction is continuously carried out in the system of composite cementitious materials, and the generation of more gelling substances such as calixarenes and calcium silicate hydrate is conducive to enhancement of the strength of specimen [10], which is consistent with the results of the mechanical test and XRD analysis.

### 4.7. Microscopic Morphology of Cementitious Materials

**Figure 10:**
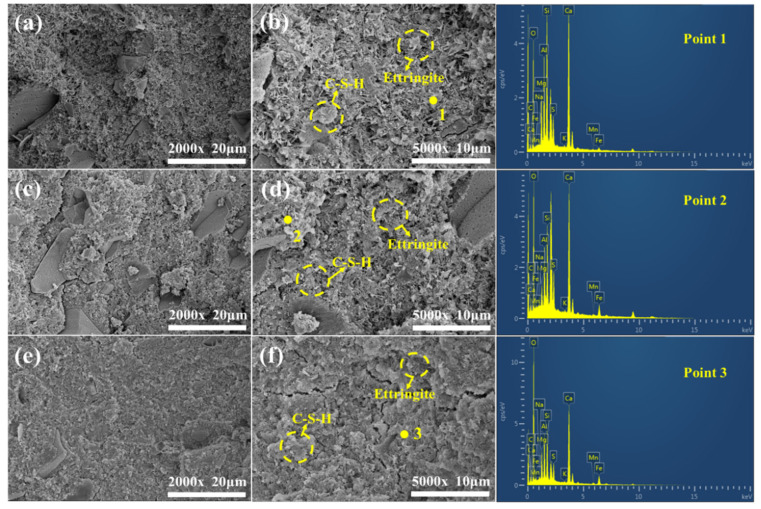
SEM-EDS plots of net slurry specimens at different age of curing: (**a**) 3 d 2000×; (**b**) 3 d 5000×; (**c**) 7 d 2000×; (**d**) 7 d 5000×; (**e**) 28 d 2000×; and (**f**) 28 d 5000×.

Figure 10 shows SEM-EDS images of the as-synthesized EMR–RM–GGBS–Ca(OH)_2_ composite cementitious materials at different age of curing. It can be seen that the microscopic morphology of the net mortar specimens maintained at different ages showed large differences. The results of elemental distribution in the yellow area in the SEM image of each sample presented in Figure 10 and Table 3 reveals that the main elements of the hydration products of each sample are Si, Ca, Al, O, and S, indicating that the main gelling substances AFt and C-S-H gels formed in samples at different ages of curing.

During the initial 3 day curing period, the specimen exhibited numerous macroscopic pores with relatively low structural density, and a substantial amount of hydration products, including AFt and C-S-H gel, is formed. The needle-shaped AFt crystals are interwoven with C-S-H gel and randomly distributed, collectively constituting the hardened skeletal structure of the specimen [37]. Due to the presence of internal pores in the specimen, the amount of gel-like substances generated by the hydration reaction was limited. Consequently, although the specimen exhibited certain mechanical properties at early stages, its strength remained relatively low. After 7 days of curing, compared to the 3 day curing period, the specimen still contained numerous pores, but the number of macroscopic pores was significantly reduced. This is primarily attributed to the continuous hydration reaction producing substantial amounts of AFt and C-S-H gel, which progressively filled the pores, thereby enhancing the specimen density and improving its mechanical properties. Upon reaching 28 days of curing, the specimen demonstrated high density with a smooth and even surface, indicating near completion of the hydration reaction, as macroscopic pores had essentially disappeared. However, microscopic examination revealed fine cracks on the specimen’s surface. This phenomenon can be explained by the fact that, during the hydration process, the abundant pores provided space for the formation and growth of AFt crystals. As the reaction progressed, these pores were filled with cementitious materials such as AFt and C-S-H gel. While this increased the structural density of the specimen, the formation and growth of ettringite led to spatial constraints, generating internal stresses that ultimately resulted in the formation of microcracks.

Si, Ca, Al, O, and S in the energy spectrum analysis indicate that C-S-H and calomel were formed at the beginning of the conservation period, and the C-S-H was in a state of amorphous agglomeration covering the surface of the material. There was also C, which may have been Ca(OH)_2_ that had not reacted completely, in the conservation age, Ca(OH)_2_ reaction with CO_2_ in the air generated CaCO_3_ so that the material has the presence of C elements. The presence of CaCO_3_ can be used as a filler to fill in the interior of the material, which resists external pressures and tensions, giving the material better compressive and tensile strength.

### 4.8. Setting Time Analysis

**Figure 11:**
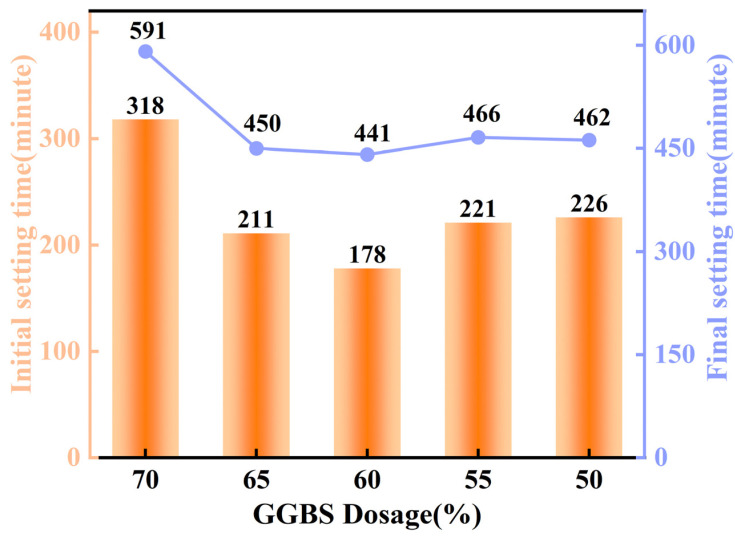
GGBS dosing and setting time.

Figure 11 shows the setting time of the as-synthesized EMR–RM–GGBS–Ca(OH)_2_ composite cementitious materials with a constant EMR dosage of 20% usage, a gradually decreasing addition of GGBS (from 70% to 50%), and a gradual increasing dosage of RM (0% to 20%). This demonstrates that the initial setting time of the EMR–RM–GGBS–Ca(OH)_2_ material was gradually shortened from 318 min to 178 min; moreover, the final setting time decreased from 591 min to 441 min as the addition amount of GGBS reduced from 70% to 60%. This proves that the addition of GGBS effectively shortened the setting time of the as-synthesized EMR–RM–GGBS–Ca(OH)_2_ composite cementitious materials, which is an excellent way to promote hydration [38]. The addition of GGBS significantly reduced the setting time, primarily due to the participation of an appropriate amount of red mud in the hydration reaction, which substantially accelerated the hydration process, thereby decreasing the setting time of the cementitious material [39]. However, when the GGBS content was significantly reduced to 50%, the relatively high proportion of red mud (RM) introduced excessive amounts of low-activity alkali metal components into the system, resulting in an extension of the initial setting time from 178 min to 226 min and increasing the final setting time from 441 min to 462 min [40].

### 4.9. Toxicity Leaching Test

**Figure 12:**
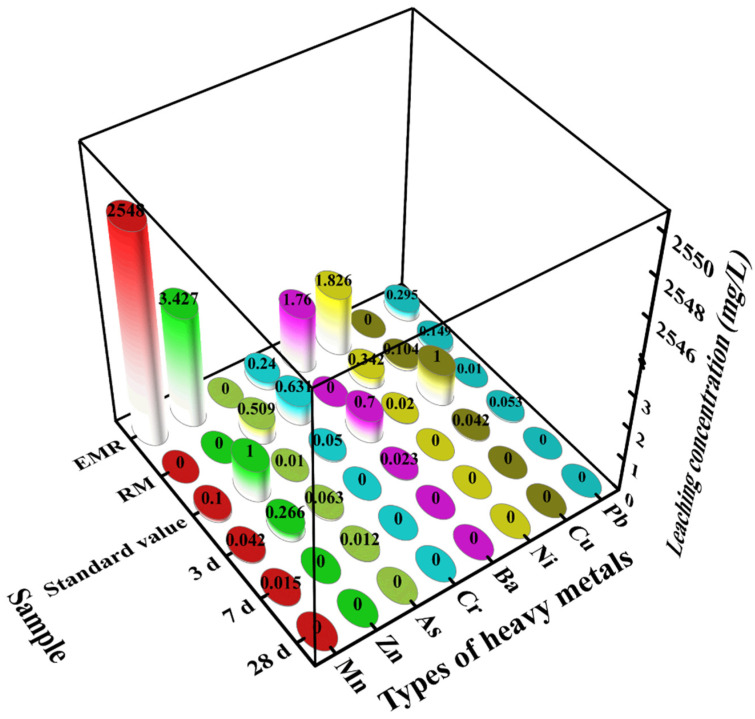
Toxic leaching concentrations for EMR and RM at different curing ages.

The study aimed to prepare cementitious materials from industrial solid wastes and the ultimate goal of being used in the construction industry or as reclamation materials. Therefore, the impact of composite cementitious materials on the environment is crucial. The test refers to the Solid Waste—Extraction Procedure for Leaching Toxicity—Sulphuric Acid and Nitric Acid Method [41] for heavy metal toxic leaching of composite cementitious materials. The environmental safety of the prepared composite cementitious materials was evaluated using the Standards for Drinking Water Quality [42]. The leachate was tested, and the results are shown in Figure 12. As, Cr, Ni, and Pb in the raw red mud and Mn, Zn, Ni, Cr, Cr, Ba, and Pb in the raw electrolytic manganese residue exceeded the standard limits. The main pollutant in the electrolytic manganese residue was Mn. After preparation of the composite cementitious materials, the concentration of its individual elements decreased significantly, and after 3 d of curing the concentration of the other elements in the specimen were lower than the standard limit values, except for As and Pb. When curing time was extended to 28 d, the elements could not be detected. Thus, the experiments show that the composite cementitious material specimens can effectively solidify and stabilize the harmful substances in RM and EMR, which is mainly attributed to the good adsorption and solidification ability of these cementitious materials [43].

## 5. Conclusions

In order to increase the utilization rate of industrial solid waste and reduce the environmental pressure caused by industrial solid waste, this study prepared composite cementitious material using industrial solid waste such as RM, EMR, and GGBS. We systematically researched the effects of RM dosage, alkali exciter calcium hydroxide dosage, and water-binder ratio on the compressive and flexural properties of electrolytic manganese residue–red mud–ground blast furnace slag–calcium hydroxide composite cementitious materials. The sample material microstructures were characterized by techniques such as XRD, SEM, EDS, TG-DTG, and FT-IR. The main products in the hydration process were analyzed, the hydration mechanism was elaborated, and finally a toxicity leaching analysis was carried out in order to verify whether this kind of cementitious material would bring extra pressure on the environment. The following conclusions were drawn based on the experimental results:

(1) Through optimization of the preparation process of composite cementitious materials, the best preparation process conditions were obtained: the optimal ratio of electrolytic manganese slag was 20%, that of red mud was 15%, that of ground blast furnace slag was 52%, that of calcium hydroxide was 13%, and the optimal water–cementitious ratio was 0.5. Under these conditions, the compressive and flexural strengths of the specimens after 28 days of curing were 27.9 MPa and 7.5 MPa, respectively.
(2) The hydration products in the composite cementitious material are mainly AFt and C-S-H gel, and the RM and EMR mixed in suitable proportions effectively stimulated the activity of GGBS. The combined effect of alkali in red mud and SO_4_^2−^ in EMR, as well as alkali excitation, further promoted the participation of active substances such as SiO_2_ and Al_2_O_3_ in the composite gelling system in the hydration reaction to generate more AFt and C-S-H gels.
(3) By conducting toxic leaching experiments on the composite cementitious material, we found that the composite cementitious material had a significant effect on the curing of heavy metals. The concentrations of all the heavy metals in the leachate of the samples at the age of 28 d did not exceed the standard limit values of the Hygienic Standard for Drinking Water (GB 5749-2022). The results show that the composite gelling system can effectively solidify and stabilize the harmful substances in electrolytic manganese slag and red mud, reducing the harm to the environment, and is thus an environmentally friendly cementitious material.

In summary, the composite cementitious material prepared from three types of industrial solid waste (RM, EMR, and GGBS) and the alkali activator calcium hydroxide not only possesses good performance, but also effective immobilizes harmful ions in the raw materials and reduces hazards to the environment, making it a green and environmental-friendly cementitious material.

## Figures and Tables

**Table 1:** Raw material XRF analysis table (% by mass).

Raw Material	CaO	SiO_2_	SO_3_	Al_2_O_3_	Fe_2_O_3_
GGBS	49.32	30.84	-	16.32	0.487
RM	2.64	4.17	-	22.08	63.74
EMR	17.07	22.96	36.00	1.77	13.08

**Table 2:** Test proportions of composite cementitious materials.

Specimen ID	Mass/%				Water-Binder Ratio
	EMR	RM	GGBS	Ca(OH)_2_	
1	20	5	65	10	0.5
2	20	10	60	10	0.5
3	20	15	55	10	0.5
4	20	20	50	10	0.5
5	20	25	45	10	0.5
6	20	15	61	4	0.5
7	20	15	58	7	0.5
9	20	15	52	13	0.5
10	20	15	49	16	0.5
11	20	15	46	19	0.5
12	20	15	52	13	0.55
13	20	15	52	13	0.6
14	20	15	52	13	0.65
15	20	15	52	13	0.7

**Table 3:** Elemental distribution results of the yellow area in the SEM image of each sample/%.

Area	C	O	Na	Mg	Al	Si	S	K	Ca	Mn	Fe
**1**	5.25	60.83	0.56	4.03	5.69	8.00	1.97	0.15	12.88	0.14	0.52
**2**	13.00	61.85	1.07	1.64	3.51	4.26	2.24	0.16	9.90	0.19	2.18
**3**	9.80	68.19	0.64	2.79	3.53	3.50	1.38	0.13	7.66	0.26	2.12

## Data Availability

The original contributions presented in the study are included in the article. Further inquiries can be directed to the corresponding authors.
